# Changes in depressive symptoms associated with spousal loss in six country context

**DOI:** 10.1093/geroni/igag101

**Published:** 2026-09-18

**Authors:** Shekhar Chauhan, Dawn C Carr

**Affiliations:** Claude Pepper Center, Florida State University, Tallahassee, Florida, United States; Department of Sociology, Florida State University, Tallahassee, Florida, United States

**Keywords:** Widowhood, Mental health, International comparative research, Longitudinal analysis

## Abstract

**Background and Objectives:**

This exploratory study examines changes in depressive symptoms following spousal loss relative to remaining married across countries, whether these associations vary by national context and gender, and the extent to which selection into widowhood accounts for cross-national differences.

**Research Design and Methods:**

This study uses data from the Health and Retirement Study international family of studies and evaluates change in depressive symptoms across six country contexts: United States, England, Mexico, China, and two combinations of European Union countries categorized by welfare state: Conservative and Social/Democratic. We evaluated change in depressive symptoms among individuals who were married at Wave 1 (2015/2016 depending on country) and at Wave 2 (2018) when some remained married while others reported a spousal loss. The analytic sample included adults aged 60 years and older (Wave 1 mean age = 69.13 years, *SD* = 6.78).

**Results:**

Using ordinary least squares regression and propensity score methods to account for selection into widowhood, results suggest that the experience of widowhood is generally similar across country contexts, with widows experiencing, on average, about 0.5 *SDs* increase in depressive symptoms (marginal effect = 0.53, *p* < .001; propensity adjusted score = 0.56, *p* < .001), a likely clinically relevant increase, accounting for selection. These effects were roughly the same for men and women in all country contexts.

**Discussion and Implications:**

Future research is needed to identify factors that may buffer the consequences of widowhood and explore cross-national interventions that may help reduce these effects.

Innovation and Translational Significance: This study advances aging research by providing one of the first harmonized longitudinal cross-national evaluations of depressive symptom change following spousal loss across diverse country contexts. Using comparable aging surveys and causal modeling approaches, we demonstrate that widowhood produces a clinically meaningful increase in depressive symptoms regardless of national policy environment or gender. The findings translate directly to practice and policy by highlighting bereavement as a universal mental health risk requiring routine screening, early psychosocial intervention, and targeted support within health and aging services. Results inform scalable international strategies to identify and support older adults during vulnerable post-bereavement transitions.

Spousal relationships are among the most central and influential social ties in later life and often serve as the primary source of emotional support, companionship, shared identity, and instrumental care as individuals age (Irani et al., 2022; Ryan et al., 2014). A large and growing body of research in aging and life course scholarship has consistently demonstrated that close social relationships are strongly associated with multiple dimensions of well-being, including mental health, happiness, and life satisfaction (Abaei & Martin, 2023, 2026; Chen & Feeley, 2014). Marital and partner relationships are especially important because of their intensity, long duration, and the high level of interdependence that develops between spouses over time. Recent empirical studies have further highlighted that well-being within couples is closely interconnected, such that one partner’s emotional state can significantly shape the other partner’s psychological outcomes (Kamp Dush & Taylor, 2012). Evidence from longitudinal data, including the Health and Retirement Study, shows that partners’ happiness and emotional well-being are strongly linked within older couples, reflecting meaningful patterns of mutual influence (Abaei & Martin, 2023, 2026). These findings suggest that spousal relationships are not only individually protective but also relationally embedded sources of well-being in later life. Given this central role, disruption of the marital bond through spousal loss represents one of the most significant life transitions in later adulthood, with important consequences for mental health and overall well-being.

Spousal loss is one of the most well-studied stressful life events in later life (Carr & Jeffreys, 2011; Holmes & Rahe, 1967; Umberson et al., 1992). The death of a spouse is particularly likely to contribute to increased depressive symptoms, which can have physiological health consequences for a surviving spouse (Domingue et al., 2021). Furthermore, spousal loss has been primarily attributed to a variety of individual factors, such as loneliness and increased burdens related to everyday chores, activities, and financial responsibilities (Utz et al., 2004; Vedder et al., 2024). On the other hand, reduced mental health consequences of widowhood are attributed to the availability of certain resources such as family support and financial status (Zheng & Yan, 2024; Zhu et al., 2024).

Extensive research shows that elevations in depressive symptoms are a normative emotional response to spousal loss (Jadhav & Weir, 2018), but much of the research to date excludes adults in low- and middle-income countries and has not explored if these effects are universal across different welfare state systems. Cultural norms around caregiving and death may shape emotional responses to spousal loss, while institutional resources such as healthcare, long-term care, and financial protection may also influence its psychological impact. For example, in more individualistic settings such as the United States, limited formal long-term care support may increase financial and caregiving strain following widowhood compared with contexts offering stronger public support systems (Angrisani et al., 2022). In settings with near-universal healthcare and strong norms of intergenerational co-residence and filial support, such as China and Mexico, surviving spouses may experience fewer adverse mental health effects due to lower financial strain and greater availability of family-based support (Monserud & Wong, 2015; Wang et al., 2014). Similarly, although European countries all offer universal healthcare, which reduces the financial burden of end-of-life medical care, some European countries rely primarily on family-oriented caregiving (i.e., those with “conservative” welfare state models), whereas others offer more extensive public services for end-of-life care regardless of available family support (i.e., those with “social democratic” welfare state models) (Esping-Andersen, 1991; Filetti, 2025; Verbakel et al., 2023). Finally, countries such as England offer a different mix of public programs, with some means-based financial supports for formal care even while also relying on expectations of informal care from families (Lyu et al., 2024). Taken together, living in different country contexts could play a role in shaping the emotional toll of spousal loss.

Despite extensive evidence linking spousal loss to higher depressive symptoms, important limitations remain in existing literature. Much of the research relies on single-country studies or cross-sectional designs, which limits our understanding of how different institutional arrangements influence the mental health consequences of spousal loss. In addition, relatively few investigations use harmonized longitudinal data that permit direct comparison across diverse national contexts over time, limiting our ability to isolate individual characteristics based on country context and examine whether country contexts influence how men and women respond to spousal loss differently. Finally, no previous research evaluating cross-national variations in widowhood has addressed how factors like systematic differences in the characteristics of widows, such as age, gender, and health status, account for any differences in the mental health consequences of widowhood across different country contexts. The present study addresses these gaps by using a novel harmonized longitudinal dataset we developed to evaluate six countries/regions with distinct social, cultural, and policy environments. We evaluate changes in depressive symptoms over two study periods (approximately 2 years) to examine the acute increases in depressive symptoms associated with spousal loss. We also include a comparison group of continuously married individuals in each country to account for typical depressive symptoms associated with aging in the absence of the widowhood experience. To account for cultural differences in the experience of grief and reporting of mental health challenges, depressive symptoms are standardized within each country context, making it possible to evaluate normative experiences of spousal loss in one country relative to normative experiences in another. Finally, we use a causal modeling approach to test whether the characteristics of widows across different countries account for variations in the mental health consequences of widowhood.

Specifically, this exploratory study addresses the following research questions:

RQ1: How much do depressive symptoms increase in association with spousal loss relative to continuously married older adults on average across different country contexts?

RQ2: How does country context moderate the effect of widowhood on increases in depressive symptoms?

RQ2a: How do these differences vary by gender?

RQ3: How does accounting for selection into widowhood explain any country context differences in the effect of widowhood on increased depressive symptoms?

## Theoretical framework

Spousal loss represents one of the most profound and consequential life transitions in later adulthood, with well-documented implications for psychological well-being (Purrington, 2023). Marriage provides emotional support, companionship, shared routines, and identity in later life, and its disruption through widowhood can lead to increased depressive symptoms. Prior research consistently shows declines in mental health following spousal loss, though the magnitude and duration of these effects vary across individuals and contexts (Bassoli et al., 2025; Shin & Park, 2025; Victor, 2026). At the same time, growing evidence from life course and social gerontology perspectives suggests that the consequences of widowhood are not uniform, but are shaped by the broader social, personal, institutional, and policy environments in which individuals are embedded (Chauhan et al., 2026; Subramanian et al., 2008). This study is motivated by the need to move beyond single-country evidence and examine whether and how the psychological consequences of widowhood differ across diverse welfare and socio-cultural contexts.

Most widowhood-mental health studies are based on single-country data, mainly from high-income Western settings, limiting understanding of how institutional contexts shape outcomes. Cross-national comparisons are needed because widowhood may vary with welfare supports, family systems, healthcare access, and gender norms. This study addresses this by examining multiple countries to assess whether effects are universal or context-dependent.

To address this gap, we use welfare state theory (Esping-Andersen, 1991) to structure cross-national variation, grouping European countries into two clusters due to data constraints. Conservative European countries include Austria, Germany, France, Switzerland, Belgium, the Czech Republic, and Poland, characterized by earnings-related benefits and stronger reliance on family-based care. Social Democratic countries include Sweden, Denmark, Spain, Italy, and Greece, with more universalistic welfare provisions and stronger public service support. This grouping enables comparison of institutional contexts in shaping widowhood’s psychological effects.

Beyond Europe, the study includes the United States, England, Mexico, and China to capture broader institutional and cultural variation. The United States reflects a liberal welfare model with limited public provision and greater reliance on private resources. England offers a comparatively stronger public healthcare and pension system within a broadly liberal context. Mexico represents a middle-income setting with limited formal welfare and strong reliance on family support. China provides a non-Western context shaped by rapid aging, strong family norms, and evolving social protection systems, allowing comparison across transitional welfare environments.

Taken together, these comparisons show that the mental health consequences of widowhood are shaped not only by individual bereavement processes but also by formal and informal support systems that vary across contexts. Stronger welfare provision may buffer depressive symptoms through healthcare, income security, and care services, while weaker or more family-dependent systems may increase vulnerability. By integrating multiple national settings, this study provides a more comprehensive account of how institutional contexts shape mental health following spousal loss.

In this study, country context is treated as a proxy for broader institutional and sociocultural environments shaping resources after spousal loss. Variation in welfare provision, healthcare, income support, family care, and social norms may affect both exposure to stressors and access to coping resources. As a result, cross-national differences in depressive symptoms following widowhood may reflect these contextual conditions.

## Data and methods

### Study design and sample

Our dataset was created using Gateway to Global Aging Data, which includes standard measures that produce harmonizable datasets with the Health and Retirement Study and related longitudinal aging surveys worldwide. To avoid potential bias in the widowhood experience that was unique to the global COVID-19 pandemic, we selected data from all country-level studies with two longitudinal study waves prior to 2020 and included well-established measures for depressive symptoms (see the Measures section below for details). We selected a sample that included respondents who were married in the first wave and then either: (a) reported that their spouse died by the second wave, or (b) remained continuously married during both study waves. We also restricted our sample to include only adults aged 60 and older to ensure we captured widowhood when this life event is most likely to occur.

We identified a total of five major longitudinal studies from international family of studies that met our inclusion criteria: United States (the Health and Retirement Study), China (China Health and Retirement Longitudinal Study), England (English Longitudinal Study of Aging), Mexico (Mexican Health and Aging Study), and the European nations in the Survey of Health, Aging and Retirement in Europe (SHARE). The SHARE data include data drawn from 27 different European Union countries. However, these countries represent diverse welfare state structures. Consequently, informed by the Esping-Andersen (1991) framework, we identified two welfare state country groups with sufficient samples of respondents who reported spousal loss during the study period: (a) Conservative European countries—which includes Austria, Germany, France, Switzerland, Belgium, Czech Republic, and Poland, which emphasize earnings-related benefits and family-based care supports; and (b) Social Democratic European countries—which includes Sweden, Denmark, Spain, Italy, and Greece, which generally include more universalistic welfare provisions and expanded public services.

We addressed missing data by using a complete-case approach. The sample was restricted to respondents married at baseline who either remained married or became widowed between waves; all other marital statuses were excluded by design. Respondents with missing outcome or covariate information were then excluded, yielding the final analytic sample. This approach ensures consistency across models but assumes missingness is random conditional on observed variables, and potential selection differences are noted as a limitation. Widowed respondents are those who experienced spousal loss between waves, with continuously married respondents serving as the comparison group. The final sample includes 4,761 individuals from the United States (*n = *4,533 continuously married and *n = *228 widows) using data from the 2016 and 2018 study waves, 3,557 individuals from England (*n = *3,457 continuously married and *n = *100 widows) using data from the 2016 and 2018 study waves, 2,989 individuals from Mexico (*n = *2,798 continuously married and *n = *191 widows) using data from the 2015 and 2018 study waves, 2,926 individuals from China (*n = *2,802 continuously married and *n = *124 widows) using data from the 2015 and 2018 study waves, 3,710 individuals from Conservative European Union countries (*n = *3,595 continuously married and *n = *115 widows), and 4,026 individuals from Social Democratic European Union Countries (*n = *43,867 continuously married and *n = *159 widows) using data from the 2014 and 2016 study waves (the 2018 wave did not include the full panel and could not be used). The total sample size includes 21,969 individuals (*n = *21,052 continuously married and *n = *917 widows). Although the number of widowed respondents is smaller than the number of continuously married respondents, this reflects the relatively low incidence of spousal loss during the observation period. Regression-based estimation is appropriate for unequal group sizes, and all models adjust for relevant covariates to ensure comparability between groups. Statistical inference is therefore based on modeled differences rather than raw group sizes.

Each dataset contributes a distinct national or regional context to the analysis of depressive symptom changes following spousal loss. The HRS, ELSA, MHAS, CHARLS, and SHARE studies were harmonized to ensure comparability across measures and designs. The United States and England represent high-income settings with different welfare and healthcare systems, while Mexico and China provide middle-income contexts with distinct family structures and social protection arrangements. SHARE enables comparison across European contexts with varying welfare-state orientations. Together, these datasets allow assessment of whether widowhood effects are consistent across diverse institutional settings.

### Measures

#### Dependent variable

*Depressive symptoms* are measured in each study based on a standard measure. The SHARE survey uses the 12-item EURO-D scale. All other surveys use versions of the Center for Epidemiologic Studies Depression scale: 10 items in China, 9 items in Mexico, and 8 items in the United States and England, with items not fully identical across countries. To ensure cross-national comparability, depressive symptom scores at each wave were standardized within each country using *z*-scores. This transformation produced values centered at the country-specific mean for adults aged 60 and older and scaled in *SD* units. The standardized score at Wave 1 was subtracted from the standardized score at Wave 2 to create a standardized change score (the outcome measure using in regression models), with all models controlling for Wave 1 to account for differences at baseline. Positive values indicate increased depressive symptoms, whereas negative values indicate reduced symptoms between the two waves. Depressive symptoms were measured using harmonized versions of the Center for Epidemiologic Studies Depression (CES-D) scale or EURO-D scale, and these scales are appropriate to use for cross-country analysis (Courtin et al., 2015; Yu et al., 2025).

#### Key variables of interest

We control for *marital status group* using a dichotomous measure (1 = became widowed, 0 = continuously married). *Country context* is a six-item measure for country context category: 1 = United States, 2= England, 3 = Mexico, 4 = China, 5 = European Union (Conservative), 6 = European Union (Social/Democratic).

Covariates were selected based on prior research demonstrating their association with both widowhood and mental health outcomes in later life. Demographic characteristics, socioeconomic resources, family support, and physical health have been shown to influence psychological well-being and may also shape individuals’ capacity to adapt to major life stressors such as spousal loss (Chauhan et al., 2026; Das et al., 2024; Tang et al., 2023; Yue & Yoon, 2025). Accordingly, age, gender, educational attainment, number of living children, functional limitations, chronic health conditions, and baseline depressive symptoms were included as control variables to reduce potential confounding in the association between widowhood and changes in depressive symptoms.

#### Control variables

We include control measures for a series of sociodemographic and health-related factors that were harmonized so that they could be measured the same across all six country contexts. *Female* is a dichotomous measure (1 = female). *Education* is measured using three categories based on educational credentials: 1 = less than high school, 2 = high school/some college, and 3 = college. *Age* is a continuous measure (range: 60–99). *Number of Living Children* is a continuous measure (range: 0–8).

We include several measures for health status given their association with mental health in later life. Each measure is drawn from both study waves to account for health decline that may otherwise explain any changes in depressive symptoms. Activities of Daily Living (ADL) and Instrumental Activities of Daily Living (IADL) limitations are included as indicators of physical functioning and independence in later life, both of which are closely linked to vulnerability to widowhood and psychological well-being. These measures capture the ability to perform basic and instrumental daily activities and are widely used indicators of functional health in aging research. Prior research has consistently shown that poorer functional health is associated with higher depressive symptoms (Ormel et al., 2002) and reduced ability to cope with major life stressors, including spousal loss. In the context of widowhood, functional limitations may also reflect broader health and caregiving needs that shape both exposure to and recovery from bereavement-related distress. Therefore, adjusting for ADL and IADL limitations helps account for preexisting differences in physical functioning that may confound the relationship between widowhood and changes in depressive symptoms across country contexts. *Number of Activity of Daily Living Limitations* is a count of limitations in bathing, eating, dressing, walking across a room, and getting in and out of bed (range: 0–5). *Number of Instrumental Activities of Daily Living Limitations* is a count of difficulties with managing money, shopping, preparing meals, and taking medications (range: 0–4). *Number of chronic conditions* is based on the number of conditions that have been diagnosed by a doctor, including stroke, arthritis, diabetes, heart disease, and high blood pressure (range: 0–5). We have also controlled for baseline depressive symptoms in our regression models. Table S3 provides details on the cross-country harmonization of all the control variables that we have used in this study.

### Analytic approach

We started by evaluating the overall characteristics of our sample differentiated by spousal loss group. We used bivariate tests (χ^2^ for categorical and *t*-tests for continuous) to examine group differences across all study variables (see Table 1), and separately evaluated differences among widows across all country contexts (see Table S1).

**Table 1:** Overall characteristics of the study sample by marital status group.

Variable	Continuously married (*n *= 21,052)		Became widowed (*n *= 917)		*p*
	*M* (*SD*)	Proportion	*M* (*SD*)	Proportion	
Depressive symptoms (W1) (standardized)	−0.01 (0.99)		0.30 (1.15)		***
Depressive symptoms (W2) (standardized)	−0.03 (0.98)		0.72 (1.25)		***
**Control variables**					
Female		0.47		0.72	***
Education					
Less than high school		0.45		0.57	***
High school/some college		0.35		0.31	***
College		0.20		0.12	***
Age (years)	69.13 (6.78)		72.92 (7.96)		***
Living children	2.87 (1.85)		3.36 (2.43)		***
**Health status**					
ADL limitations	0.21 (0.68)		0.27 (0.76)		**
ADL limitations (W2)	0.25 (0.78)		0.42 (1.04)		***
Instrumental ADL limitations	0.13 (0.52)		0.21 (0.63)		***
Instrumental ADL limitations (W2)	0.18 (0.63)		0.34 (0.83)		***
Chronic conditions	1.45 (1.12)		1.73 (1.14)		***
Chronic conditions (W2)	1.62 (1.16)		1.92 (1.16)		***

*Note*. ADL = activities of daily living; results provide the mean (continuous measures) or proportion (dichotomous measures). All control measures are drawn from the baseline study wave unless otherwise specified. The table presents differences in characteristics between continuously married respondents and those who transitioned to widowhood between waves. Significance is based on bivariate analyses using chi-square tests (categorical) and *t-*tests (continuous).

Standard error significance:

* *p *< .05.

** *p *< .01.

*** *p *< .001.

Next, to address RQ1, we use Ordinary Least Square (OLS) regression models (see Table S2) to evaluate the association between spousal loss group and change in depressive symptoms, controlling for country context and all other control variables (Model 1). To address RQ2, we add the interaction between country context and spousal loss group. To address RQ2a, we examine these effects for men and women using separate regression models for each group.

Finally, to address RQ3, we use inverse probability regression models. These models include two equations: one predicting selection into the spousal loss group by country (which involves creating a 12-category measure capturing both marital status groups across each country context) and a regression model predicting change in depressive symptoms (the same as our OLS models), which accounts for selection based on the propensity weight. This approach allows us to simulate a randomized controlled study, comparing multiple “treatment” groups at once. The *ipwra* Stata program computes values for the “potential outcome means” (POMEAN) (i.e., the predicted change in depressive symptoms) for each of the 12 groups, accounting for the observable factors determined to select them into the widowhood-country context category. We also use these models to calculate the “average treatment effects” (ATE), or the average difference between an assigned control group and all other “treatment” groups. We evaluate how selection influences differences in the effect of spousal loss across country contexts by calculating the predicted change scores for each spousal loss group in each country. To explore whether observable differences explain differences by country and marital status group, we calculate both our results using OLS regression alone and our results from our propensity score models. To calculate effect sizes for each approach (with and without accounting for selection), we use the post-estimation *margins* command in Stata to calculate the estimated change in depressive symptoms for each group in our OLS models, and to calculate these values when accounting for selection in our *ipwra* models, we use POMEAN and ATE results. Results are shown in Table 2. Due to small sample sizes, it is not possible to evaluate gender differences using propensity score models. Model specification for the selection model was based on systematic evaluation of all available control measures to identify the most parsimonious combination of measures, which provided robust results across alternative model specifications and did not violate the overlap assumption. The final measures used in those models are provided in Table S1.

**Table 2:** Predicted depressive symptoms by marital status, relative to other country contexts.

Country context	Marital category	Predicted # Symp	Significant difference relative to other contexts					
			United States	England	Mexico	China	EU (conservative)	EU (social/democratic)
**Marginal effects results**								
United States	Widowed	0.60***	n/a	NS	NS	***	NS	*
	Married	0.01	n/a	NS	**	***	NS	NS
England	Widowed	0.51***	NS	n/a	NS	***	NS	*
	Married	0.03*	NS	n/a	***	***	NS	*
Mexico	Widowed	0.47***	NS	NS	n/a	***	NS	***
	Married	−0.06***	**	***	n/a	NS	**	+
China	Widowed	−0.02	***	***	***	n/a	***	***
	Married	−0.06***	***	***	NS	n/a	**	*
EU (conservative)	Widowed	0.46***	NS	NS	NS	***	n/a	**
	Married	0.00	NS	NS	**	**	n/a	NS
EU (social/democratic)	Widowed	0.88***	*	*	***	***	**	n/a
	Married	−0.02	NS	*	+	*	NS	n/a
**Propensity score adjusted results**								
United States	Widowed	0.70***	n/a	NS	NS	**	NS	NS
	Married	0.01	n/a	NS	***	**	NS	NS
England	Widowed	0.64***	NS	n/a	NS	*	NS	NS
	Married	0.00	NS	n/a	***	**	NS	NS
Mexico	Widowed	0.52***	NS	NS		+	NS	NS
	Married	−0.12***	***	***		NS	***	**
China	Widowed	0.26**	**	*	+	n/a	NS	***
	Married	−0.14**	**	**	NS	n/a	**	*
EU (conservative)	Widowed	0.50***	NS	NS	NS	NS	n/a	NS
	Married	0.00	NS	NS	***	**	n/a	NS
EU (social/democratic)	Widowed	0.75***	NS	NS	NS	***	NS	n/a
	Married	−0.03	NS	NS	**	*	NS	n/a

*Note*. EU = European Union; n/a = not available. “Predicted # Symp” indicates predicted standard deviations above (+) or below (–) the mean, and the associated significance indicates whether this difference is significantly different from 0. Significant difference relative to other contexts indicates whether the value for each country/region is statistically different relative to other contexts.

* *p *< .05.

** *p *< .01.

*** *p *< .001; NS indicates *p *> .05.

## Results

Table 1 summarizes the characteristics of our study sample, showing overall differences by marital status groups across all country contexts. Although all characteristics are statistically different between the continuously married group and those who became widowed, on average, we observe that widows had 0.31 *SDs* higher depression than their married counterparts at Wave 1 and 0.75 *SDs* higher at Wave 2, with an increase of 0.42 *SDs* across the two time points for widows without controlling for any other factors. We also evaluated differences in the characteristics of those who experienced spousal loss across all country contexts (Table S1), finding that Wave 1 scores ranged from 0.14 (China) to 0.54 (England) *SDs* and Wave 2 scores ranged from 0.17 (China) to 1.14 (EU—Social/Democratic) *SDs* and differences over the two time points ranging from 0.03 (China) to 0.75 (EU Social/Democratic) *SDs*. Beyond depressive symptoms, several other measures suggest that the individual characteristics of widows are substantially different across countries, particularly regarding educational attainment, with some countries having very low proportions with any kind of formal educational credential, number of living children, and age being particularly different across country contexts. Samples differed across demographic and health characteristics. Widows in the United States, England, and European groups were older on average than those in Mexico and China. The proportion of women ranged from 63% in China to 81% in Social Democratic European countries. Educational attainment also varied, with higher shares of low education in Mexico and China compared with the United States, England, and Europe. Differences were also observed in the number of children, chronic conditions, and baseline depressive symptoms. Despite these differences, all contexts showed increases in depressive symptoms following widowhood, though the magnitude varied. These patterns highlight the importance of accounting for compositional differences in cross-national analyses.

To address RQ1, we used OLS regression, controlling for country context and all other factors, with results indicating an average increase of 0.53 *SDs* in depressive symptoms for widows relative to their continuously married counterparts. Marginal effects calculations (not shown) indicate this is due to a 0.51 *SD* increase for widows (*p *< .001) and a 0.02 *SD* decrease among those continuously married (*p *< .05; see Table S2). Table 2 presents adjusted marginal predictions of changes in standardized depressive symptoms for continuously married and widowed respondents across country contexts using both OLS and propensity score models. Predicted values represent expected changes between Wave 1 and Wave 2 after adjusting for covariates, with positive values indicating increases in depressive symptoms. Comparing OLS and propensity score estimates allows assessment of the extent to which differences reflect selection into widowhood. Table 2 reports these changes across contexts and shows whether estimates differ significantly across countries after accounting for selection.

To address RQ2, we included an interaction between marital status group and country context (Table S2, Model 2). Results indicate significant differences in the change in depressive symptoms by country context. The effect size calculations, based on post-estimation marginal effects calculations, are shown in Table 3. Effects range from no significant increase, on average (China), to an increase of 0.90 *SDs* (EU Social/Democratic). Despite these differences, these effects are not statistically different across countries except China, which is lower than all other countries.

**Table 3:** Total effect size for spousal loss on change in depressive symptoms, by OLS regression and propensity score-adjusted results.

Country context	Marginal effects results			Propensity score adjusted results		
	Effect size	Confidence interval		Effect size	Confidence interval	
		Min	Max		Min	Max
Overall	0.53***	0.45	0.60	0.56***	0.46	0.65
United States	0.59***	0.44	0.74	0.69***	0.43	0.95
England	0.48***	0.23	0.72	0.64***	0.39	0.89
Mexico	0.53***	0.38	0.68	0.64***	0.44	0.85
China	0.04^NS^	–0.13	0.22	0.40***	0.19	0.60
EU (conservative)	0.46***	0.27	0.65	0.50***	0.24	0.75
EU (social/democratic)	0.90***	0.70	1.10	0.78***	0.54	1.01

*Note*. EU = European Union; OLS = Ordinary Least Square. Effect size is the predicted number of standard deviations for those who become widowed relative to their continuously married counterparts.

* *p *< .05.

** *p *< .01.

*** *p *< .001; NS indicates *p *> .05.

To address RQ2a, we conducted these analyses separately for men and for women. Results are provided in Table S2, Models 3–6. We also conducted marginal effects estimates (not shown) for further evaluation. Results show that, on average, women relative to men have a larger increase in depressive symptoms in England, China, and both EU country contexts, but men have a larger increase in the United States and Mexico. However, none of the calculated differences are statistically significant across the gender groups. Overall, these results show that widowhood similarly influences men and women within all country contexts.

To address RQ3, we conducted an analysis using propensity weighting based on inverse probability weighted regression adjustment. The POMEANs are provided in Table 3. Compared with our models that did not account for selection into marital status and country context, we observe fairly small differences. Overall, the OLS regression models showed that widowhood is associated with an average increase of 0.53 *SDs* versus an increase of 0.56 *SDs* with selection. Although some countries showed relatively small increases in predicted effect sizes (United States: +0.1 *SDs*; Mexico: +0.11 *SDs*; EU (Conservative): +0.04 *SDs*; EU (Social/Democratic): −0.12 *SDs*), two countries showed relatively larger increases. The estimated effect size for England increased by 0.16 *SDs*, and the effect size for China increased the most, moving from no statistically significant increase associated with widowhood to a 0.4 *SD* increase in association with widowhood. Figure 1 shows the differences in the estimated effects for OLS versus the propensity score-based models.

**Figure 1:**
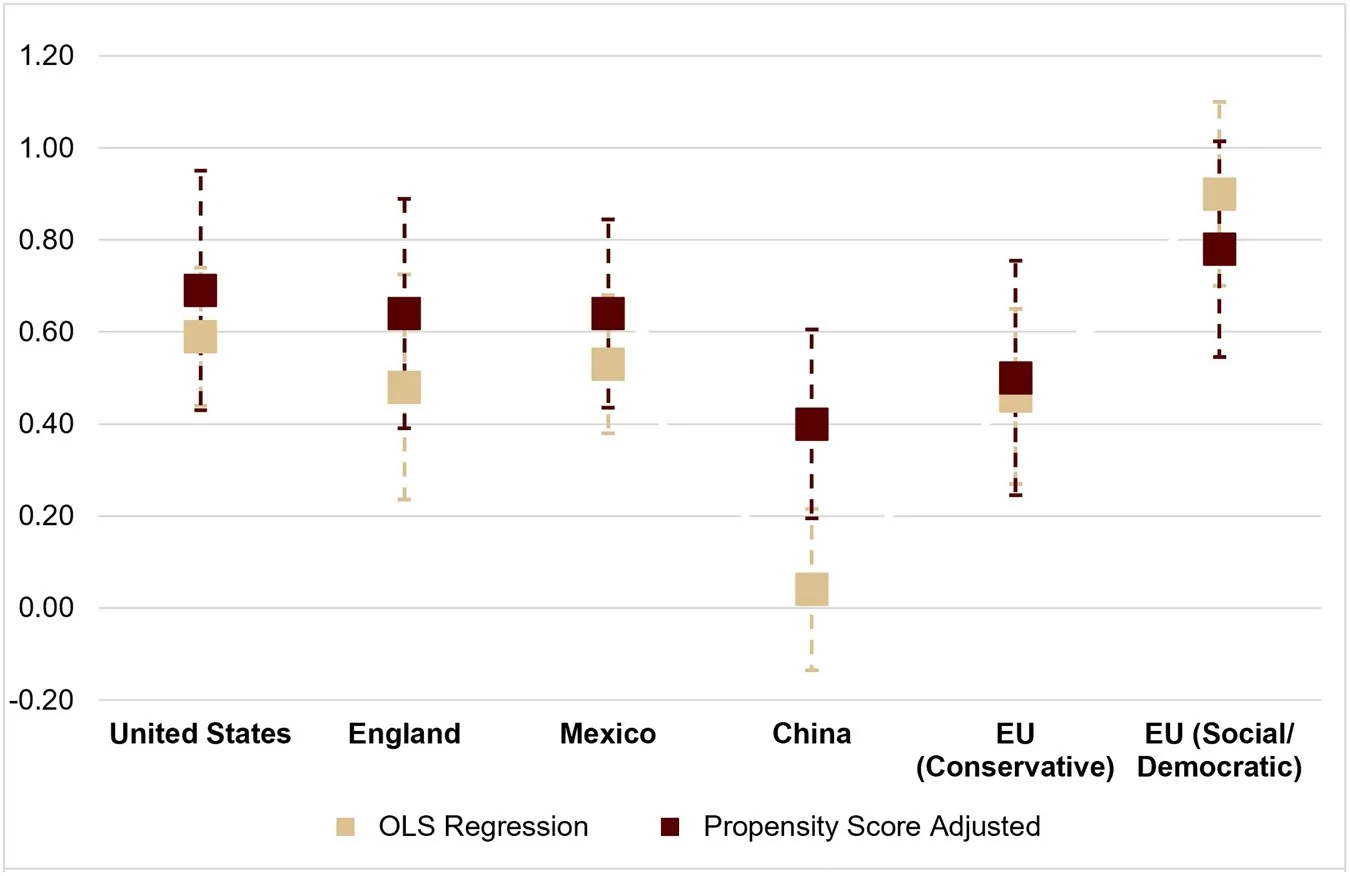
Predicted effect size of spousal loss for depressive symptoms, with and without accounting for selection effects. Error bars reflect 95% confidence intervals for each estimate. Propensity score models weight models for selection into widowhood/married country categories, including age, gender, and number of activities of daily living limitations, number of instrumental activities of daily living limitations, and number of chronic conditions at baseline. EU = European Union; OLS = Ordinary Least Square.

## Discussion

This study offers a comprehensive evaluation of the effect that spousal loss has on surviving spouses by way of increased depressive symptoms. Several decades of research have shown normative increases in depressive symptoms related to spousal loss, but these studies have typically focused on individuals within a single country (Li et al., 2023; Mu et al., 2025; Umberson et al., 1992), and the few that have examined individuals across multiple countries (e.g., Richardson et al., 2020) have not comprehensively examined longitudinal changes in depressive symptoms.

Our study included longitudinal data from four countries and two European Union regions (consisting of data from 12 countries), measuring change in depressive symptoms between Wave 1, when all individuals (60 and older) were married, and Wave 2, when some of the married individuals experienced a spousal death. Overall, our results suggest that across country contexts, spousal loss is associated with an average of about a half *SD* increase in depressive symptoms in the immediate period following widowhood. Although the specific instruments used to evaluate depressive symptoms in this study were not designed for clinical purposes, this level of increase is generally accepted as clinically relevant, suggesting a need for intervention (Pim et al., 2014).

An important goal of our study was to determine whether the emotional toll of widowhood differs across different country contexts. Although in two of the countries evaluated the difference between the two study waves was about 3 years apart (Mexico and China) versus 2 years in all other contexts, the results were generally not statistically different across country contexts. Mexico was also lower than the EU (Social/Democratic) context, and China effectively showed no increase associated with widowhood, but those differences disappeared when accounting for selection into widowhood. Although we were unable to use selection modeling strategies to evaluate differences in effects for men and women, we observed no significant gender differences in any country contexts.

Our findings both support and extend prior research on widowhood and mental health. Consistent with previous studies conducted in the United States, Europe, and Asia (Jadhav & Weir, 2018; Schaan, 2013; Schmitz, 2021), we find that spousal loss is associated with meaningful increases in depressive symptoms among older adults. However, our findings also extend the literature by demonstrating that this relationship is remarkably consistent across diverse institutional and welfare-state contexts. Although welfare-state theory suggests that more extensive social protections may buffer the adverse consequences of major life events (Bambra, 2009; Bergqvist et al., 2013), the relatively modest differences observed across country contexts suggest that the emotional consequences of losing a spouse may be less influenced by institutional environments than the relatively universal effects of this stressful life event, such as social wellbeing and caregiving-related challenges that commonly occur. This finding does not suggest that institutional contexts are unimportant. Rather, it indicates that the psychological consequences of losing an intimate partner may be broadly shared across societies. The attenuation of cross-national differences after propensity score adjustment also highlights the importance of selection processes, suggesting that individual characteristics associated with widowhood vary across countries and should be accounted for in future comparative research.

Despite the relatively universal effects observed in our final models, the findings for China highlight the importance of selection processes. In the OLS models, widowhood was not associated with a significant increase in depressive symptoms. However, after propensity score adjustment, widowhood was associated with a 0.40 *SD* increase in depressive symptoms. This suggests that characteristics associated with becoming widowed in China may mask the psychological consequences of spousal loss in unadjusted analyses. Chinese widows in our sample were younger and reported comparatively lower baseline depressive symptoms than widows in several other country contexts. Although our data do not permit direct examination of the underlying mechanisms, the findings may reflect the central role of family relationships in providing emotional, social, and instrumental support in later life, with spouses often serving as the primary source of companionship and daily support (Chen & Short, 2008; Silverstein et al., 2006). Consequently, the loss of a spouse may substantially disrupt established support networks and daily routines, increasing vulnerability to psychological distress. Our findings suggest that these consequences may be underestimated when selection into widowhood is not adequately accounted for. Despite ongoing social and demographic changes, family remains a primary source of emotional and social support for many older adults in China. The stronger effect observed after propensity score adjustment indicates that the psychological consequences of spousal loss may be greater than previously recognized.

Similarly, the findings from England underscore the importance of accounting for selection when estimating the mental health consequences of widowhood. Although conventional regression models showed a small but significant increase in depressive symptoms among continuously married respondents, this increase disappeared after propensity score adjustment, while the estimated effect of widowhood remained unchanged. Together with the findings from China, these results suggest that differences in respondent characteristics can bias conventional regression estimates and highlight the value of propensity score methods for reducing selection bias in cross-national studies of widowhood.

Another important finding is that the largest increase in depressive symptoms associated with widowhood was observed in Social Democratic European countries. Even after accounting for selection into widowhood, the estimated effect remained substantial (0.78 *SDs*). Although these countries provide extensive social welfare and health care supports, such resources may not mitigate the emotional consequences of losing a spouse, suggesting that additional interventions targeting emotional and social support are needed following bereavement.

Future research is needed to identify the mechanisms underlying stronger bereavement effects in some national contexts. Although welfare-state institutions can reduce financial strain and caregiving burdens, they may not replace the emotional companionship, daily social interaction, and social integration provided by a spouse. Moreover, higher-quality spousal relationships before bereavement may amplify the psychological consequences of the loss. From a stress process perspective, the loss of a spouse represents the loss of a primary social bond and source of emotional support, resources that are not easily substituted by formal institutions (Pearlin et al., 1990). In countries with strong welfare systems, older adults may rely less on extended family, making the marital relationship more central to daily life. Consequently, the loss of a spouse may cause greater disruption to emotional well-being and social connectedness. Although we cannot directly test these mechanisms, our findings suggest that institutional support and intimate relationships operate through different pathways. While welfare systems may reduce financial and health-related disadvantages, they may also make the emotional consequences of spousal loss more pronounced. Further, the persistence of emotional losses may simply be less amenable to institutional intervention, even in settings where material and health-related needs are comparatively well protected (Bambra, 2009; Eikemo et al., 2008).

More broadly, the comparison between the OLS and propensity score results highlights the importance of accounting for selection into widowhood. Individuals who become widowed differ systematically from those who remain married in age, health, gender, and family characteristics. While conventional regression adjusts for these differences, propensity score methods further balance observed characteristics, providing estimates that more closely approximate the counterfactual of remaining married. The reduction in cross-national differences after propensity score adjustment suggests that part of the observed variation reflects differences in who becomes widowed rather than differences in the consequences of widowhood itself.

Overall, we conclude that although there are some small variations across country contexts, these differences are likely attributed to the characteristics of the widows within these countries. In Table S1, the differences across the different populations of widows are rather stark. Although health is an important factor that differentiates widows from their continuously married counterparts, the average age for widows in Mexico and China is about 6 years lower than in other country contexts. In addition, although women are more likely than men to become widowed, this difference is particularly pronounced in the EU (Social/Democratic) country context where 81% of widows are female versus China where 63% are female. In Mexico, widows have an average of five living children, about three fewer children than those living in the EU (both contexts) and England. These findings suggest that although the individual factors that influence whether and how much widowhood influences increases in depressive symptoms is likely fairly consistent across country contexts, the main differences across country contexts is related to the average differences in the individual characteristics of people who become widowed across contexts. That is, even though individual differences in the characteristics of widows are influenced by the institutional arrangements in those countries, people living in countries with more generous social welfare state designs do not seem to be protected relative to those living in countries with the least supportive welfare designs.

A unique contribution of this study is the use of harmonized longitudinal data across multiple country contexts combined with propensity score methods to account for selection into widowhood. Our findings show that, although the magnitude of the association varies across contexts, widowhood is consistently associated with increased depressive symptoms after accounting for differences in the characteristics of those who become widowed. These results highlight the need for timely mental health screening and supportive interventions for recently widowed older adults across diverse national settings.

Study limitations should be noted. Two countries have a longer inter-wave period (3 vs 2 years), which may allow more recovery time after widowhood. Depressive symptom measures also differ across countries; although within-country standardization was used, residual comparability issues may remain. Harmonized data provide scale scores rather than item-level data, preventing reliability testing such as Cronbach’s alpha. The two-wave design captures short-term change only and not longer-term trajectories. Finally, cultural factors influencing both widowhood experiences and depressive symptoms reporting could not be directly measured and warrant future research. A further limitation is that the study does not distinguish respondents by duration of widowhood. Although individuals classified as widowed experienced spousal loss between survey waves, the harmonized data do not allow consistent identification of time since bereavement across countries. As a result, the estimates reflect average changes in depressive symptoms following the transition to widowhood and may mask heterogeneity in short-term and longer-term psychological responses. Future research should examine how the effects of widowhood vary by duration since spousal loss across different contexts.

Our study is limited by the available measures across all harmonized studies, which may exclude important observable factors that were available to use in our models. Future research will be needed to address these limitations and expand on our results to evaluate differences across widows living in different countries. Further, previous research using cross-sectional data suggests that certain resources may buffer mental health consequences of widowhood in some countries but not in others (Chauhan et al., 2026). Finally, because marital status was measured at discrete survey waves, we could not capture all marital transitions occurring between interviews. As a result, respondents who experienced spousal loss and subsequently remarried before follow-up would not be classified as widowed. Although remarriage is relatively uncommon in this age group, this limitation should be considered when interpreting the findings.

We conclude that well-established findings showing significant mental health consequences associated with spousal loss seem to be relatively universal across a wide range of country contexts, with relatively similar results for men and women. Future research is needed to explore universal and country-specific factors that may buffer mental health consequences of widowhood and identify potential interventions to reduce the mental health consequences of widowhood. Further research is needed to identify mechanisms underlying cross-national differences in the mental health consequences of widowhood. While this study documents variation across contexts, the roles of social support, family structure, economic resources, and access to services remain unclear. Future work should also examine longer-term trajectories of depressive symptoms and potential sources of resilience following bereavement. Expanding harmonized cross-national analyses to additional countries may further clarify how social and institutional contexts shape adaptation to widowhood in later life.

## Research and policy implications

These findings suggest that widowhood, regardless of country context, presents a significant inflection point in elevating risks of depression. The relatively consistent 0.5 *SD* increase in depressive symptoms associated with widowhood across diverse national contexts suggests that bereavement-related mental health problems are not restricted to specific welfare state environments or country-contexts. This suggests that all public health systems would benefit from incorporating routine bereavement screening, short-term psychosocial interventions, and community-based supports into primary care and aging services to mitigate acute mental health deterioration following spousal loss. Because cross-national differences are related primarily to the characteristics of those who become widowed, spousal bereavement interventions that focus on high-risk individuals may work similarly across all country contexts.

Beyond policy, these findings have important implications for clinical practice and service delivery. Healthcare providers should recognize widowhood as a period of heightened vulnerability to depressive symptoms and consider routine screening in the years following spousal loss, along with referral to appropriate mental health and social support services when needed. Community organizations and aging service providers can also help reduce isolation by promoting social connectedness among recently widowed older adults.

More broadly, this study highlights the value of harmonized longitudinal data and propensity score methods for studying widowhood across diverse contexts. Future research should further examine mechanisms underlying cross-national differences, including social support, family networks, cultural expectations, and access to formal care, to better understand how institutional and social environments shape adaptation to bereavement.

## Supplementary Material

igag101_Supplementary_Data

## Data Availability

The data underlying this article were obtained from the Gateway to Global Aging Data website (G2G Aging) and are available to registered users at https://g2aging.org/harmonized-data/get-data. The authors do not have permission to redistribute the underlying data. The analytic methods used in this study are described in the manuscript, and the materials necessary to understand and replicate the analyses are available from the corresponding author upon reasonable request. Access to country-specific datasets may be subject to additional data-use agreements and restrictions imposed by the respective data providers. This study was not preregistered.
