# Supplementary material for: Changes in depressive symptoms associated with spousal loss in six country context
Source: Innov Aging. 2026 Sep 18;10(11):igag101. doi: 10.1093/geroni/igag101 (PMC13695514; doi:10.1093/geroni/igag101)
Supplement: Supplementary file 1 [file igag101_supplementary_data.docx]

***Innovation in Aging* Supplementary Material: Chauhan & Carr. Changes in depressive symptoms associated with spousal loss in six country contexts.**

Supplementary Table 1. Baseline characteristics of widows by country context

|  | **United States** | **England** | **Mexico** | **China** | **EU**  **(Conservative)** | **EU**  **(Social/Dem)** | ***p*** |
| --- | --- | --- | --- | --- | --- | --- | --- |
|  | **Proportion/**  **Mean (SD)** | **Proportion/**  **Mean (SD)** | **Proportion/**  **Mean (SD)** | **Proportion/**  **Mean (SD)** | **Proportion/**  **Mean (SD)** | **Proportion/**  **Mean (SD)** |  |
| Depressive symptoms (W1) (standardized) | 0.19  (1.17) | 0.54  (1.35) | 0.24  (1.14) | 0.14  (1.00) | 0.48  (1.10) | 0.39  (1.09) | * |
| Depressive symptoms (W2) (standardized) | 0.72  (1.35) | 0.81  (1.44) | 0.68  (1.09) | 0.17  (1.11) | 0.75  (1.12) | 1.14  (1.23) | *** |
| Female ^a^ | 0.70 | 0.71 | 0.71 | 0.63 | 0.70 | 0.81 | * |
| Education |  |  |  |  |  |  | *** |
| Less than high school | 0.15 | 0.43 | 0.93 | 0.89 | 0.43 | 0.67 |  |
| High school/some college | 0.63 | 0.44 | 0.01 | 0.10 | 0.38 | 0.23 |  |
| College | 0.22 | 0.13 | 0.06 | 0.01 | 0.19 | 0.10 |  |
| Age ^a^ | 74.41  (8.01) | 74.61  (8.28) | 69.38  (6.78) | 68.75  (6.22) | 75.50  (8.06) | 75.38  (7.15) | *** |
| # Living children | 3.30  (1.93) | 2.37  (1.58) | 5.41  (2.70) | 3.32  (1.65) | 2.43  (1.69) | 2.29  (1.35) | *** |
| # ADL limitations ^a^ | 0.21  (0.58) | 0.20  (0.47) | 0.34  (0.85) | 0.37  (0.86) | 0.34  (0.94) | 0.22  (0.78) |  |
| # ADL limitations (W2) ^a^ | 0.35  (0.90) | 0.34  (0.82) | 0.45  (1.13) | 0.48  (1.16) | 0.47  (1.08) | 0.43  (1.14) |  |
| # Instrumental ADL limitations ^a^ | 0.15  (0.48) | 0.15  (0.50) | 0.19  (0.66) | 0.25  (0.56) | 0.36  (0.89) | 0.19  (0.68) |  |
| # Instrumental ADL limitations (W2) | 0.26  (0.64) | 0.32  (0.85) | 0.25  (0.70) | 0.45  (0.94) | 0.50  (1.08) | 0.40  (0.89) | * |
| # Chronic conditions ^a^ | 2.16  (1.09) | 1.56  (1.00) | 1.51  (1.06) | 1.16  (1.11) | 1.85  (1.71) | 1.82  (1.16) | *** |
| # Chronic conditions (W2) | 2.29  (1.12) | 1.74  (1.02) | 1.67  (1.07) | 1.44  (1.20) | 2.10  (1.21) | 2.05  (1.16) | *** |

Note: ADL = activity of daily living. The samples of widows include: United States, *N =* 228; England, *N =* 100; Mexico, *N =* 191; China, *N =* 124; EU (Conservative), *N =* 115; EU (Social/Dem), *N =* 159. Measures provided were identified as significant predictors associated with selection into marital status-country context categories. The *p*-value indicates significant differences in characteristics across country contexts based on a chi-square test for female and based on ANOVA test results for all others: *** *p* <0.001; **p* <0.05.

^a^ Indicates that the measure was used in the propensity score model.

Supplementary Table 2. OLS regression models evaluating standardized change in depressive symptoms for men and women aged 60+ years old by country context

|  | **Total** | | **Male** | | **Female** | |
| --- | --- | --- | --- | --- | --- | --- |
|  | **Model 1** | **Model 2** | **Model 3** | **Model 4** | **Model 5** | **Model 6** |
|  | **Coeff**  **(RSE)** | **Coeff**  **(RSE)** | **Coeff**  **(RSE)** | **Coeff**  **(RSE)** | **Coeff**  **(RSE)** | **Coeff**  **(RSE)** |
| Became Widowed | 0.53***  (0.04) | 0.59***  (0.08) | 0.46***  (0.07) | 0.67***  (0.14) | 0.56***  (0.05) | 0.56***  (0.09) |
| Country Context |  |  |  |  |  |  |
| England | 0.01  (0.02) | -0.02  (0.02) | -0.03  (0.02) | -0.02  (0.02) | 0.05+  (0.03) | 0.05+  (0.03) |
| Mexico | -0.07***  (0.02) | -0.07**  (0.02) | -0.10***  (0.03) | -0.11***  (0.03) | -0.05  (0.03) | -0.03  (0.03) |
| China | -0.10***  (0.02) | -0.08***  (0.02) | -0.10***  (0.03) | -0.08**  (0.03) | -0.12**  (0.04) | -0.08*  (0.04) |
| EU (Conservative) | -0.01  (0.02) | -0.01  (0.02) | -0.04+  (0.02) | -0.03  (0.02) | 0.01  (0.03) | 0.01  (0.03) |
| EU (Social/Dem) | -0.02  (0.02) | 0.03  (0.02) | -0.06*  (0.02) | -0.05*  (0.02) | 0.01  (0.03) | -0.01  (0.03) |
| England x Widowed |  | -0.11  (0.15) |  | -0.35  (0.25) |  | -0.05  (0.18) |
| Mexico x Widowed |  | -0.06  (0.11) |  | 0.08  (0.21) |  | -0.13  (0.13) |
| China x Widowed |  | -0.55***  (0.12) |  | -0.75***  (0.18) |  | -0.42**  (0.16) |
| EU Cons x Widowed |  | -0.13  (0.12) |  | -0.34  (0.23) |  | 0.05  (0.15) |
| EU Social/Dem x Widowed |  | 0.31*  (0.13) |  | -0.13  (0.24) |  | 0.41**  (0.15) |
| Female | 0.11***  (0.01) | 0.11***  (0.01) |  |  |  |  |
| Education |  |  |  |  |  |  |
| High school/some college | -0.08***  (0.01) | -0.08***  (0.01) | -0.08***  (0.02) | -0.08***  (0.02) | -0.08***  (0.02) | -0.08***  (0.02) |
| College | -0.14***  (0.02) | -0.13***  (0.02) | -0.11***  (0.02) | -0.11***  (0.02) | -0.17***  (0.03) | -0.16***  (0.03) |
| Age | 0.00  (0.00) | 0.00  (0.00) | 0.00  (0.00) | 0.00  (0.00) | 0.00  (0.00) | 0.00  (0.00) |
| # Living children | 0.01+  (0.00) | 0.01+  (0.00) | 0.01*  (0.00) | 0.01*  (0.00) | 0.00  (0.01) | 0.00  (0.01) |
| # Depressive symptoms | -0.54***  (0.01) | -0.54***  (0.01) | -0.56***  (0.01) | -0.56***  (0.01) | -0.52***  (0.01) | -0.52***  (0.01) |
| # ADL limitations | -0.02  (0.01) | -0.02  (0.01) | -0.03  (0.02) | -0.03  (0.02) | -0.01  (0.02) | -0.01  (0.02) |
| # ADL limitations (W2) | 0.14***  (0.01) | 0.14***  (0.01) | 0.17***  (0.02) | 0.17***  (0.02) | 0.10***  (0.02) | 0.11***  (0.02) |
| # Instrumental ADL limitations | -0.06***  (0.02) | -0.06***  (0.02) | -0.04  (0.03) | -0.04  (0.03) | -0.08**  (0.03) | -0.08**  (0.03) |
| # Instrumental ADL limitations (W2) | 0.14***  (0.02) | 0.14***  (0.02) | 0.13***  (0.02) | 0.13***  (0.02) | 0.15***  (0.02) | 0.15***  (0.02) |
| # Chronic conditions | -0.02  (0.01) | -0.02  (0.01) | -0.04*  (0.02) | -0.04*  (0.02) | 0.00  (0.02) | 0.01  (0.02) |
| # Chronic conditions (W2) | 0.09***  (0.01) | 0.09***  (0.01) | 0.09***  (0.02) | 0.09***  (0.02) | 0.08***  (0.02) | 0.08***  (0.02) |
| Constant | -0.88  (0.07) | -0.08  (0.07) | -0.08  (0.08) | -0.10  (0.08) | 0.01  (0.11) | 0.03  (0.11) |
| Akaike Information Criterion | 53,497 | 53,432 | 26,044 | 26,016 | 27,158 | 27,122 |

Note: ADL = activity of daily living. Statistical significance indicators show associations with depressive symptoms: **p* <0.05; ** *p* <0.01; *** *p* <0.001.

Supplementary Table 3. Description of the variables across datasets used in the analysis

**1. CESD**

| **Question** | **CHARLS**  **(2015 & 2018)** | **HRS**  **(2016 & 2018)** | **MHAS**  **(2015 & 2018)** | **ELSA**  **(2016 & 2018)** |
| --- | --- | --- | --- | --- |
| Variable Coding | How you felt and behaved during the last week  (1. Rarely or none of the time (less than 1 day)  2. Some or a little of the times ( 1 or 2 days)  3. Occasionally or a moderate amount of the time (3 or 4 days)  4. Most or all of the time (5 – 7 days)) | Much of the time during the past week,  (Yes/No) | These questions refer to how you have felt during the past week. For each question please tell me if the majority of the time...  (Yes/No) | Now think about the past week and the feelings you have experienced. Please tell me if each of the following was true for you much of the time during the past week.  (Yes/No) |
| CONCENTRATION | I had trouble keeping my mind on what I was doing |  |  |  |
| DEPRESSED | I felt depressed | You felt depressed. | You felt depressed? | You felt depressed |
| TIRED OR LOW ENERGY |  |  | You felt you had a lot of energy? |  |
| FEELING FEARFUL | I felt fearful |  |  |  |
| FEELING ALONE | I felt lonely | You felt lonely | You felt lonely? | You felt lonely |
| BOTHERED BY THINGS | I was bothered by things that don’t usually bother me |  |  |  |
| EVERYTHING AN EFFORT | I felt everything I did was an effort | You felt that everything you did was an effort. | You felt that everything you did was an effort? | You felt that everything you did was an effort |
| HOPEFUL FUTURE | I felt hopeful about the future |  |  |  |
| HAPPY | I was happy | You were happy | You felt happy? | You were happy |
| RESTLESS SLEEP | My sleep was restless | Your sleep was restless | You felt your sleep was restless? | Your sleep was restless |
| ENJOYED LIFE |  | You enjoyed life | You felt that you enjoyed life? | You enjoyed life |
| FELT SAD |  | You felt sad | You felt sad? | You felt sad |
| FELT TIRED |  |  | You felt tired? |  |
| FELT UNMOTIVATED | I could not get ”going” | You could not get going |  | You could not get going |
| CRONBACH’S ALPHA |  |  |  |  |

**2. SHARE EURO-D 12-ITEM SCALE**

| **S.No.** | **Variable coding** | **Wording** | **Type** |
| --- | --- | --- | --- |
| 1. | MH002_Depression | In the last month, have you been sad or depressed? | Yes/No |
| 2. | MH003_Hopes | What are your hopes for the future? | Any hopes mentioned/No hopes mentioned |
| 3. | MH004_WishDeath | In the last month, have you felt that you would rather be dead? | Any mention of suicidal feelings or wishing to be dead/ No such feelings |
| 4. | MH005_Guilt | Do you tend to blame yourself or feel guilty about anything? | Obvious excessive guilt or self-blame/ No Such Feelings/Mentions guilt or self-blame, but it is unclear if these constitute obvious or excessive guilt or self-blame (a3) |
|  | IF MH005_Guilt = a3;  MH006_BlameForWhat | So, for what do you blame yourself? | Example(s) given constitute obvious excessive guilt or self-blame/ Example(s) do not constitute obvious excessive guilt or self-blame, or it remains unclear if these constitute obvious or excessive guilt or self-blame |
| 5. | MH007_Sleep | Have you had trouble sleeping recently? | Trouble with sleep or recent change in pattern/ No sleep troubling |
| 6. | MH008_Interest | In the last month, what is your interest in things? | Less interest than usual mentioned/ No mention of loss of interest/ Non-specific or uncodeable response |
|  | IF MH008_Interest = a3; MH009_KeepUpInt | So, do you keep up your interests? | Yes/No |
| 7. | MH010_Irritability | Have you been irritable recently? | Yes/No |
| 8. | MH011_Appetite | What has your appetite been like in the last month? | Diminution in desire for food/No diminution in desire for food/Non-specific or uncodeable response |
|  | IF MH011_Appetite = a3;  MH012_EatMoreLess | So, have you been eating more or less than usual? | Less/ More/ Neither more nor less |
| 9. | MH013_Fatigue | In the last month, have you had too little energy to do the things you wanted to do? | Yes/ No |
| 10. | MH014_ConcEnter | How is your concentration? For example, can you concentrate on a television programme, film or radio programme? | Difficulty in concentrating on entertainment/No such difficulty mentioned |
|  | MH015_ConcRead | Can you concentrate on something you read? | Difficulty in concentrating on reading/No such difficulty mentioned |
| 11. | MH016_Enjoyment | What have you enjoyed doing recently? | Fails to mention any enjoyable activity/Mentions ANY enjoyment from activity |
| 12. | MH017_Tear | In the last month, have you cried at all? | Yes/No |

**3. Marital Status Group**

| Note: We have used harmonized variable as available in respective harmonized dataset, which is coded as follows: | | |
| --- | --- | --- |
| CHARLS (2015 & 2018) | 1. Married 1. Partnered 2. Separated 3. Divorced 7. Widowed 8. Never Married | In each of the survey waves, the variable was coded as provided in a column before. For the analysis, we dichotomized the variable 1=became widowed and 0= continuously married. Became widowed are those who were married at the first wave but got widowed in the follow-up wave. Continuously married are those people who were recorded as married in both the waves used in the study. |
| HRS (2016 & 2018) | 1. Married 2. Married, Spouse absent 3. Partnered 4. Separated 5. Divorced   7. Widowed  8. Never Married |  |
| MHAS (2015 & 2018) | 1. Married 3. Partnered 4. Separated 5. Divorced 7. Widowed 8. Never Married |  |
| ELSA 92016 & 2018) | 1. Married 3. Partnered 4. Separated 5. Divorced   7. Widowed  8. Never Married |  |
| SHARE (2014 & 2016) | 1. Married 3. Partnered 4. Separated 5. Divorced   7. Widowed  8. Never Married |  |

**4. Female**

In each of the harmonized datasets, the gender variable is provided as a dichotomous variable named as ragender where 1= man and 2=woman. We have recoded this variable as female where 0=male and 1=female.

**5. Education**

| **CHARLS**  **(2015 & 2018)** | We have used a categorical variable from harmonized dataset named as **raeduc_c**. The variable has 10 categories named as follows:  1. No formal education/ illiterate  2. Did not finish primary school but cap  3. Sishu  4. Elementary school  5. Middle school  6. High school  7. Vocational school  8. Two/Three Year College/Associate degree  9. Four Year College/Bachelor's degree  10. Post-graduate (master/PhD) | We categorized this variable into three categories for this study:  1= Less than high school (combining categories 2, 3, 4, and 5)  2= High School/ some college (combining categories 6, 7, and 8) – for ease, we call this category as high school in the manuscript draft.  3= College (combining categories 9 and 10) |
| --- | --- | --- |
| **HRS**  **(2016 & 2018)** | We have used a categorical variable from harmonized dataset named as **raeduc**. The variable has 5 categories named as follows:   1. Lt High School 2. GED 3. High School Graduate 4. Some College 5. College and Above | We categorized this variable into three categories for this study:  1= Less than high school (combining categories 1 and 2)  2= High School/ some college (combining categories 3 and 4) – for ease, we call this category as high school in the manuscript draft.  3= College (category 5)  **Note:** we dropped some cases who had 0 years of education (variable name **raedyrs**) but were falling under any three categories that we created. |
| **MHAS**  **(2015 & 2018)** | We have used a categorical variable from harmonized dataset named as **raedisced**. This variable follows ISCED classification. The variable has 6 categories named as follows:   0. Less than primary education 1. Primary education 2. Lower secondary education 3. Upper secondary education 4. First stage of tertiary education 5. Second stage of tertiary education | We categorized this variable into three categories for this study:  1= Less than high school (combining categories 0,1, and 2)  2= High School/ some college (category 3) – for ease, we call this category as high school in the manuscript draft.  3= College (categories 4 and 5) |
| **ELSA**  **(2016 & 2018)** | We have used a categorical variable from harmonized dataset named as **raeduc_e**. The variable has 5 categories named as follows:   1. Lt High School 3. High School Graduate 4. Some college 5. College and Above | We categorized this variable into three categories for this study:  1= Less than high school (category 1)  2= High School/ some college (combining categories 3 and 4) – for ease, we call this category as high school in the manuscript draft.  3= College (category 5)  **Note:** Apart from this, we have another variable named **raedyrs_e** that represents the age of the respondents for their educational attainment. Accordingly, we have reassigned some cases based on the corresponding age and educational degree. |
| **SHARE (2014 & 2016)** | We have used a categorical variable from harmonized dataset named as **raedisced**. This variable follows ISCED classification. The variable has 7 categories named as follows:   0. None 1. Primary education 2. Lower secondary education 3. Upper secondary education 4. Post-secondary non tertiary education 5. First stage of tertiary education 6. Second stage of tertiary education | We categorized this variable into three categories for this study:  1= Less than high school (combining categories 1 and 2)  2= High school/ some college (categories 3 and 4) – for ease, we call this category as high school in the manuscript draft.  3= College (categories 5 and 6) |

**6. Age**

Age is a continuous variable available in each separate harmonized dataset

**7. Number of Living Children**

Number of living children is available is each separate harmonized dataset as a continuous variable

**8. Activity of Daily Limitations**

The respective harmonized dataset provided a continuous five-item ADL limitation scale that we used in this study. The commonly used ADLs are ***dressing, walking, bathing, eating, and getting in and out of bed***. Respondents in all the surveys were asked to exclude any difficulties that they expect to last less than three months. The measure is consistent across the surveys.

The exact wording of the questions was almost similar across the survey. The exact wording was “Please tell me if you have any difficulty with these activities because of a physical, mental, emotional or memory problem. Exclude any difficulties you expect to last less than three months”- Dressing, including putting on shoes and socks, walking across a room, Bathing or showering, Eating, such as cutting up your food, and Getting in or out of bed.

**9. Instrumental Activity of Daily Living**

| **Question** | **CHARLS (2016)** | **HRS (2016)** | **MHAS (2016)** | **SHARE (2014)** | **ELSA (2016)** |
| --- | --- | --- | --- | --- | --- |
|  | Please tell me whether you have difficulty performing any of the following tasks on a regular basis. Exclude any difficulties that you expect to last less than three months  ***Because of health and memory problems, do you have any difficulties with*** | Please tell me whether you have any difficulty with each activity I name. If you don't do the activity at all, just tell me so. Exclude any difficulties that you expect to last less than three months  ***Because of a health or memory problem do you have any difficulty with*** | Please tell me if you have any difficulty with the activities that I mention to you. If you do not do any of the following activities, simply tell me.  Do not include difficulties that you believe will last less than three months.  **Because of a health problem, do you have any difficulty** | Please tell me if you have any difficulty with these activities because of a physical, mental, emotional or memory problem. Exclude any difficulties you expect to last less than three months. | Here are a few everyday activities. Please tell me if you have any difficulty with these because of a physical, mental, emotional or memory problem. Exclude any difficulties you expect to last less than three months. |
| ***USING A TELEPHONE*** | making phone calls | making phone calls |  | Making telephone calls | Making telephone calls |
| ***TAKING MEDICINE*** | Taking medications | Taking medications | taking medications? | Taking medications | Taking medications |
| ***HANDLING MONEY*** | managing your money, such as paying your bills, keeping track of expenses, or managing assets | Managing your money -- such as paying your bills and keeping track of expenses | managing your money? | Managing money | Managing money, such as paying bills and keeping track of expenses |
| ***SHOPPING*** | shopping for groceries | shopping for groceries | shopping for groceries | Shopping for groceries | Shopping for groceries |
| ***PREPARING MEAL*** | preparing hot meals | preparing a hot meal | preparing a hot meal? | Preparing a hot meal | Preparing a hot meal |
| **WORK** |  |  |  | Doing work around the house or garden | Doing work around the house or garden |
| **GETTING AROUND** |  | using a map to figure out how to get around in a strange place |  | Using a map to figure out how to get around in a strange place |  |

Note. Since MHAS survey has asked for IADL using only four items. We created a harmonized 4-item IADL scale ranging between 0-4. The items include are difficulty with medicine, managing money, shopping for groceries, and preparing a hot meal.

**10. Number of Chronic Conditions**

Each harmonized dataset included information on respondents' chronic health conditions. We utilized five chronic conditions that were measured consistently across datasets: stroke, arthritis, diabetes, hypertension, and heart problems. These conditions were combined to create a continuous measure of chronic disease burden ranging from 0 to 5, with higher values indicating a greater number of chronic conditions reported by the respondent. The exact wording of the questions was almost similar across the survey. The exact wording was “Has any health professional ever told you that you have” - Hypertension or high blood pressure, Diabetes or high blood sugar, Chronic heart diseases such as Coronary heart disease (heart attack or Myocardial Infarction), congestive heart failure, or other chronic heart problems, Stroke, and Arthritis or rheumatism, Osteoporosis or other bone/joint diseases.
